# Interleukin 17 Promotes Expression of Alarmins S100A8 and S100A9 During the Inflammatory Response of Keratinocytes

**DOI:** 10.3389/fimmu.2020.599947

**Published:** 2021-02-12

**Authors:** Carolin Christmann, Stefanie Zenker, Leonie Martens, Janina Hübner, Karin Loser, Thomas Vogl, Johannes Roth

**Affiliations:** ^1^ Institute of Immunology, University of Muenster, Muenster, Germany; ^2^ Department of Dermatology, University of Muenster, Muenster, Germany; ^3^ Department of Human Medicine, Institute of Immunology, Faculty VI - Medicine and Health Sciences, University of Oldenburg, Oldenburg, Germany

**Keywords:** S100A8, S100A9, myeloid-related proteins, MRP8, MRP14, calprotectin, keratinocytes, psoriasis

## Abstract

Psoriasis is one of the most common immune-mediated inflammatory skin diseases. Expression and secretion of two pro-inflammatory molecules of the S100-alarmin family, S100A8 and S100A9, in keratinocytes is a hallmark of psoriasis, which is also characterized by an altered differentiation of keratinocytes. Dimers of S100A8/S100A9 (calprotectin) bind to Toll-like receptor 4 and induce an inflammatory response in target cells. Targeted deletion of S100A9 reduced the inflammatory phenotype of psoriasis-like inflammation in mice. A role of S100-alarmins in differentiation and activation of keratinocytes was suggested but has been never shown in primary keratinocytes. We now confirm that induction of S100-alarmins in an imiquimod-induced murine model of psoriasis-like skin inflammation was associated with an increased expression of interleukin (IL)-1α, IL-6, IL-17A, or TNFα. This association was confirmed in transcriptome data obtained from controls, lesional and non-lesional skin of psoriasis patients, and a down-regulation of S100-alarmin expression after IL-17 directed therapy. However, analyzing primary S100A9^−/−^ keratinocytes we found that expression of S100A8/S100A9 has no significant role for the maturation and inflammatory response pattern of keratinocytes. Moreover, keratinocytes are no target cells for the pro-inflammatory effects of S100A8/S100A9. However, different cytokines, especially IL-17A and F, highly abundant in psoriasis, strongly induced expression of S100-alarmins preferentially during early maturation stages of keratinocytes. Our data indicate that expression of S100A8 and S100A9 does not primarily influence maturation or activation of keratinocytes but rather represents the inflammatory response of these cells during psoriasis.

## Introduction

Psoriasis is one of the most common immune-mediated inflammatory skin diseases with a prevalence of 2% to 3% in western industrial countries. The disease is characterized by epidermal thickening due to hyperkeratosis, infiltration of immune cells in dermis and epidermis, parakeratosis, and neovascularization. Psoriasis has a multifactorial genesis with a genetic predisposition. It can be triggered by various environmental factors, including injury, infection, and medications (1). Immunological dysfunction in psoriasis represents an overlap between auto-immunity and auto-inflammation. The cross-talk between immune cells and their corresponding cytokines with keratinocytes has been shown to play an important role in the pathogenesis of psoriasis, especially the T_H_1- and IL-23/T_H_17 axis (2).

Two pro-inflammatory molecules of the S100-family, S100A8 [myeloid-related protein 8 (MRP 8), calgranulin A] and S100A9 (MRP 14, calgranulin B), are highly up-regulated in keratinocytes and leukocytes of psoriatic skin whereas they cannot be found in the skin of healthy controls. S100A8 and S100A9 belong to the S100-family of calcium-binding proteins. S100-proteins show a tissue-specific expression pattern and have versatile functions including regulation of cell signal transduction, cell differentiation, cell motility, apoptosis, or modulation of membrane–cytoskeletal interactions. Some members exhibit also extracellular effects (3–5). S100A8 and S100A9 are expressed in high amounts in neutrophils and monocytes but not in mature tissue macrophages, dendritic cells, or lymphocytes. In addition, they are induced in keratinocytes during dermal inflammation. S100A8 and S100A9 form non-covalently associated heterodimeric S100A8/S100A9 complexes, also known as calprotectin, which represent the physiologically relevant form of these proteins (6). S100A8/S100A9 are released during cellular stress or tissue damage and act as so-called damage-associated molecular pattern molecules (DAMPs) or alarmins (7). Extracellular S100A8/S100A9 trigger inflammatory response patterns in innate immune cells *via* activation of TLR4-dependent signaling mechanisms (8, 9). S100A8 and S100A9 are the most abundant DAMPs in many inflammatory conditions, like infections, auto-immune diseases, rheumatoid arthritis, or inflammatory bowel disease. Due to their local expression and release at site of inflammation S100A8/S100A9 are reliable biomarkers for monitoring inflammatory disease activity (5, 10–12). Knock-out of the S100A8/S100A9 axis leads to attenuation of disease activity in many murine models of inflammation (8, 13).

During dermal inflammation or wound healing complexes of S100A8 and S100A9 are expressed and released by keratinocytes and activated leukocytes especially in psoriasis lesions (13–17). Serum levels of S100A8/S100A9 show a close correlation to disease activity in psoriasis patients (18). Moreover, S100A8 and S100A9 are located in the psoriasis susceptibility locus 4 (19). Several genes of the S100-family and proteins involved in epidermal cornification form a so-called epidermal differentiation complex on human chromosome 1q21 (20).

Targeted deletion of S100A9 in a psoriasis model induced by keratinocyte-specific deletion of transcriptional regulator Jun resulted in a strongly attenuated inflammation which was at least partly mediated by expression of complement factor C3 (15). Accordingly, expression of dominant active S100A8-dimers in a model of TNFα-driven psoriasis-like dermatitis accelerated skin inflammation leading to an aggravated phenotype in mice. These mice developed severe psoriasis-like skin inflammation, including scaling, hyperproliferation, and de-differentiation of keratinocytes with thickened epidermis (acanthosis), loss of the granular layer, extension of the cornified envelope (parakeratosis), and hyperpigmentation of the basal layer resulting in a high adapted Psoriasis Area and Severity Index (PASI) within a few days after birth. The mice showed dermal expression of IFN-γ, IL-6, IL-17, IL-22, IL-23, and IL-36 in the skin, which is an inflammatory pattern typical for psoriasis (6). However, the specific role of S100A8/S100A9 expression in keratinocytes during the process of psoriasis has never been addressed.

Comparing primary wildtype and S100A9^−/−^ keratinocytes we now demonstrate that expression of S100A8/S100A9 by keratinocytes has surprisingly no significant effects on the maturation of these cells nor on the inflammatory response pattern induced by various triggers in keratinocytes. Extracellular S100A8/S100A9 is also no major stimulus for inflammatory activation of keratinocytes in contrast to IL-1α, IL-17A, IL-17F, TNFα, or flagellin-mediated stimulation. However, especially IL-17A and F strongly induced S100A8 and S100A9 expression preferentially during early maturation of keratinocytes indicating that expression and release of these DAMPs is an additional factor during IL-17 driven dermal inflammation.

## Methods

### Animals

C57BL/6- and BALB/c wildtype (WT) mice were obtained from Harlan laboratories (Germany) or Charles River (Germany). S100A9^−/−^ mice were generated as described previously and backcrossed to at least F12 generation (21). Animals were used at the age of 8–20 weeks. All experiments with mice were performed with the approval by the local ethics committee, in accordance with the German regulations of the Society for Laboratory Animal Science (GV-SOLAS), the European Health Law of the Federation of Laboratory Animal Science Associations (FELASA), and by the State Review Board, office for the environment, nature, and municipal affairs North-Rhine-Westphalia, Germany, according to the German law for animal welfare (LANUV).

### Imiquimod-Induced Psoriasis-Like Skin Inflammation

The psoriasis-like skin inflammation was performed in BALB/c or C57BL/6 mice as described previously (22). Briefly, mice were treated daily with imiquimod (IMQ) cream (5% Aldara, Meda Pharma; daily dose of 4.17 mg of the active compound) on a defined shaved back area for 7 consecutive days. Control mice were treated similarly with an equal amount of a vehicle basic cream (DAC—German Pharmaceutical Codex, local pharmacy). The course of disease was monitored daily. Inflammatory parameters and histological changes were analyzed 24 h after the last IQM treatment.

### Psoriasis Area and Severity Index

To assess disease severity of the IMQ-treated mice, a clinical score was applied which was based on the Psoriasis Area and Severity Index (PASI) used for human patients. Since the affected area in mice is always the same, only the three main parameters of psoriasis were scored, namely erythema, thickness, and scaling. A scale of 0–5 was used as a cumulative score of all parameters, wherein score 0 indicates no signs of disease and score 5 represents strong inflammation with scales and lesions.

### Cells and Cell Culture Conditions

Isolation of primary epidermal keratinocytes was performed as described earlier (23). Briefly, disinfected tail skins of adult mice were digested overnight at 4˚C with trypsin (0.25%; w/o CaCl_2_, Gibco, Thermo Fisher Scientific, USA). Detached epidermal cells were seeded overnight at 32°C, 5% CO_2_ in minimum essential medium with Earle`s Balanced Salt Solution medium (Lonza, Switzerland) containing 0.2 mM CaCl_2_ (Merck, Germany). Subsequently 1x10^6^ isolated cells were cultured on fibronectin (Roche, Switzerland)/rat tail collagen I (Becton Dickinson, Corning, USA)-coated six well-culture plates in Keratinocyte Growth Medium 2 with supplement mix (PromoCell, Germany) to the confluence of 70–80%. Culture and stimulation conditions in Keratinocyte Growth Medium 2 (PromoCell, Germany) are indicated in **Supplementary Figure 1**. Times of stimulations are indicated in figure legends. Reagents: flagellin from *Salmonella typhimurium* (100 ng/ml, InvivoGen, France), rm IL-1α, rm IL-17A, rm IL-17F, and rm TNFα (100 ng/ml, BioLegend, USA), S100A8 (8). Possible endotoxin contaminations of S100A8 proteins were evaluated by a sensor chromogenic LAL endotoxin assay (GenScript, USA) and were < 2 pg/µg protein.

### Differentiation and Stimulation Protocol of Naïve Primary Keratinocytes *Ex Vivo*

Isolated primary naïve keratinocytes were cultured on fibronectin/collagen-coated culture plates in normal growth medium (Keratinocyte Growth Medium 2 with supplement mix; KGM) with 0.06 mM CaCl_2_ to a confluence of 70–80%. Afterward, the medium was changed to 1 mM CaCl_2_ to initiate the maturation of naïve keratinocytes. Depending on the maturation status, primary naïve keratinocytes were stimulated for 24 h in cell culture medium (Keratinocyte Growth Medium 2 w/o supplement mix (KBM) with 1 mM CaCl_2_) with flagellin from *S. typhimurium* (100 ng/ml), rm IL-1α, rm IL-17A, rm IL-17F and rm TNFα (100 ng/ml) and S100A8 (5 µg/ml). Undifferentiated (d0 of maturation): KBM media with 0.06 mM CaCl_2_ with 24 h stimulation; early matured (d1 of maturation): KBM media with 1 mM CaCl_2_ with 24 h stimulation; fully matured (d4 of maturation): KGM media with 1 mM CaCl_2_ for 72 h and afterward KBM media with 1 mM CaCl_2_ for 24 h stimulation (**Supplementary Figure 1**).

### Quantitative Real-Time PCR

Isolation of RNA from primary keratinocytes or snap-frozen mouse skin tissue was described in detail previously (6). Primary keratinocytes were harvested in lysis buffer. Snap-frozen mouse skin samples were cut and homogenized in lysis buffer. RNA was isolated using the NucleoSpin RNA Kit (Macherey-Nagel). RNA was transcribed in complementary DNA (cDNA) and used for RT-PCR. Data were acquired with the CFX 384 Real-Time System and CFX-manager software, version 3.1 (Bio-Rad, USA). Sample data are measured in duplicates, were calculated as 2–ΔΔCt and are presented as relative copies per ribosomal protein L (RPL) housekeeping gene expression. The primer pair sequences are listed in **Supplementary Table 1**.

### Microscopy

Culture and stimulation conditions are indicated in the figure legends. Immunohistochemistry of paraffin skin sections (4 µm) was performed as described earlier (6). Briefly, fixed skin tissues were prepared for common hematoxylin and eosin staining (HE). For immunohistochemistry, the S100A9 or S100A8 signal was detected using purified polyclonal rabbit anti-mouse/human S100A8 or S100A9 antibodies (working concentration 4 µg/ml and 1.58 µg/ml, respectively) (8) and the Vector ABC-AP kit (Vector Laboratories, USA). For immunofluorescence analyses, primary keratinocytes were cultured on fibronectin (Roche, Switzerland)/rat tail collagen I (Becton Dickinson, Corning, USA)-coated coated glass coverslips (VWR, Germany) to confluence. After culture and stimulation as indicated in the figure legends, cells were fixed with formaldehyde and permeabilized with Triton-X100. After blocking of unspecific binding sites with bovine serum albumin, primary polyclonal rabbit anti-mouse/human S100A8 or rabbit anti-mouse S100A9 antibodies (working concentration 2.6 µg/ml and 2.1 µg/ml, respectively) were applied overnight at 4°C followed by secondary goat anti-rabbit Alexa Fluor 546-coupled IgG antibody (8 µg/ml, Thermo Fisher Scientific, USA) for 1 h in the dark at room temperature. Sections were counterstained with DAPI (1 µg/ml, Merck, Germany) to visualize nuclei and mounted with Dako Fluorescence Mounting Medium (Dako, USA). Corresponding IgG isotype controls served as controls. All images were taken by an inverted microscope (Axio Observer, Zeiss, Germany), analyzed with the software AxioVision Rel. 4.8 (Zeiss, Germany, scale bar: 100 µm). For immunofluorescence staining lesional skin tissues were embedded in NEG-50 (Thermo Fisher Scientific) and cut into 3 µm sections. Sections were fixed with cold acetone for 5 min and blocked for 90 min with 2% BSA at room temperature. Thereafter, sections were incubated overnight at 4°C with an appropriate dilution (1:100 in antibody dilution buffer, DCS-Diagnostics, Hamburg, Germany) of primary antibodies against pan cytokeratin (rabbit polyclonal, Abcam, Cambridge, UK); Gr-1 (clone RB6-8C5, BioLegend, San Diego, CA); MPO (goat polyclonal, Bio-Techne, Minneapolis, MN); CD4 (clone RM4-5, BioLegend) or IL-17A (clone TC11-18H10.1, BioLegend). Afterward, sections were incubated for 3 h with secondary antibodies coupled to Alexa-Fluor 488 or Alexa Fluor 594 (secondary antibodies were purchased from Thermo Fisher Scientific, Waltham, MA; dilution 1:1,000). In most experiments, nuclei were counterstained with DAPI (Sigma-Aldrich, St. Louis, MI). Subsequently, slides were mounted with Kaiser’s glycerol gelatine (Merck Millipore, Burlington MA) and analyzed using an Olympus BX63 microscope and the cellSens software (Olympus, Muenster Germany). Images were imported in Photoshop CS2 (Adobe) for creating final figures (scale bar: 50 µm).

### Western Blot Analysis

Cells were lysed in M-PER^®^ Mammalian Protein Extraction reagent (Thermo Fisher Scientific, USA) containing a protease inhibitor mixture (Roche, Switzerland). Equal amounts of protein were separated on SDS polyacrylamide gels and transferred to nitrocellulose membranes. Membranes were probed with primary antibodies, namely: rabbit anti-mouse/human S100A8 (2 µg/ml) or S100A9 (1 µg/ml) (8), rabbit anti-β-actin (dilution 1:10,000, Cell Signaling Technology, USA) overnight at 4°C. Primary antibodies were detected with corresponding HRP-conjugated secondary antibodies (Cell Signaling Technology, USA) and developed with ECL.

### S100A8/S100A9 Analysis by Enzyme Linked Immuno-Sorbent Assay

Serum from WT mice and keratinocytes cell-culture supernatants were used to analyze S100A8/S100A9 levels by ELISA as described previously (24). Briefly, the ELISA system detects S100A8/S100A9 heterodimers. A polyclonal antibody against S100A8 was used as capturing antibody and a biotinylated polyclonal antibody for detection of S100A9. Results are expressed as ng/ml S100A8/S100A9. For each sample, 20 µl serum was diluted 1:50, 1:100, and 1:200 and measured in duplicate. If all values were in a linear range, the value was accepted. Otherwise, another dilution range was run to fit into the linear range. Differences between linearity in serum and buffers used for the standard curve are a major drawback in most commercial assays.

### Cytokine and Chemokine Measurements

LEGENDplex™ mouse inflammation panel and pro-inflammatory chemokine panel (each 13-plex; BioLegend, USA) was performed according to manufacturer’s instructions. Analyte measurements were done using Navios flow cytometer (Beckman Coulter, USA) and calculated *via* LEGENDplex™ data analysis software (BioLegend, USA).

### Human Gene Expression Analysis

Publically available expression data of 99 samples run on Affymetrix HU133 Plus 2.0 microarrays were downloaded from the Gene Expression Omnibus (GEO) database under accession ID GSE53552. These samples were obtained from 25 psoriasis patients’ lesional and non-lesional skin, as well as post-treatment with brodalumab as described in detail earlier (25). Analysis was performed with the limma (26) and affy (27) bioconductor packages, implemented in the R programming language. RMA was used to normalize and log2 transform the expression data. Additionally, public gene expression data of skin from psoriatic patients and normal controls with the accession ID of GSE13355 were used. This included 180 samples (CTRL = normal skin from 64 controls; PN = uninvolved skin from 58 psoriasis patients; PL = involved skin from the same psoriasis patients). The data was pre-analyzed as documented earlier (28). This included batch/sex effect correction and RMA normalization of expression values obtained from Affymetrix HU133 Plus 2.0 microarrays. Correlation between gene expression probes was calculated using the Hmisc package (29) and the resulting Spearman’s “rho” was tested for significance (p < 0.05).

### Statistical Analysis

Statistical analysis was performed by using GraphPad Prism 5 (GraphPad Software, USA). Data were presented as mean ± SEM if not indicated otherwise. Statistical significance was analyzed by unpaired two-sided Student’s t-test or ANOVA. Significant p-values were denoted by asterisks (*p < 0.05, **p < 0.01, ***p < 0.001). Differential expression analysis was performed with the limma (28) package and genes were found to be differentially expressed between conditions with a p-value of 0.05 after adjustment for multiple testing (Benjamini and Hochberg) and a fold change of at least 2.

## Results

### S100A8 and S100A9 Are Up-Regulated During Imiquimod-Induced Psoriasis-Like Skin Inflammation

**Figure 1:**
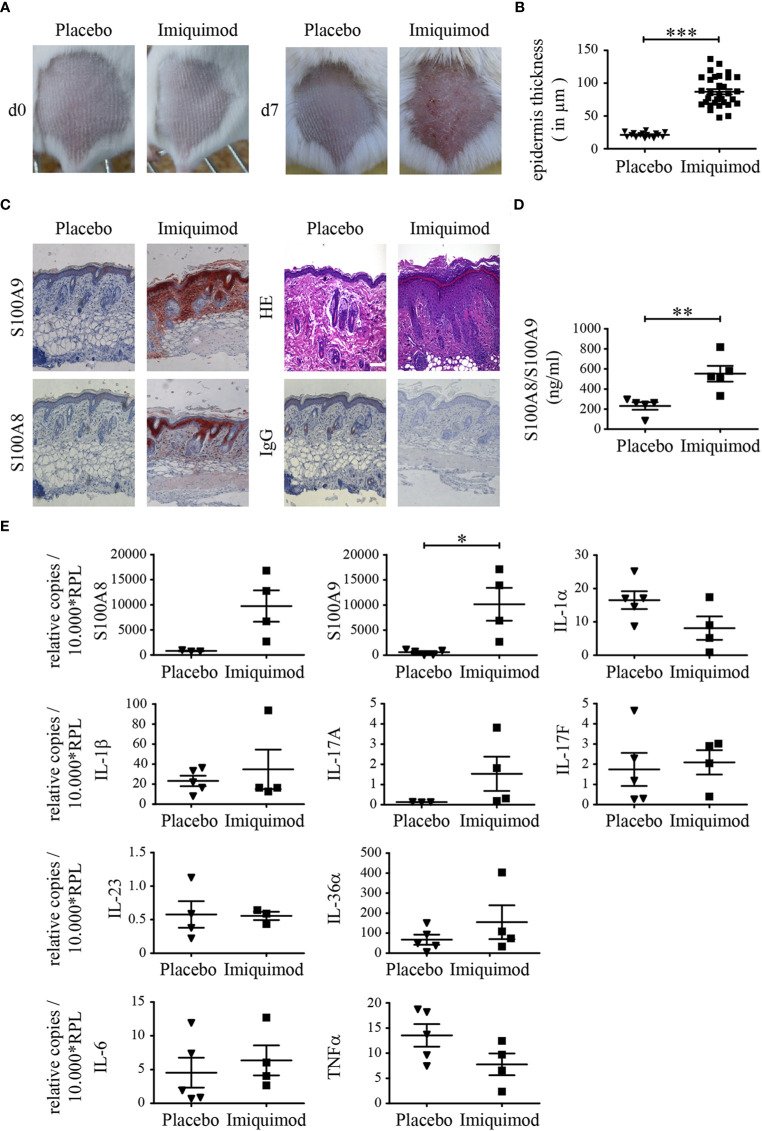
S100A8 and S100A9 are up-regulated in psoriasis like skin inflammation. Naïve BALB/c mice were treated with imiquimod (IMQ) or placebo cream for 7 consecutive days. **(A)** Typical skin appearance of IMQ- or placebo cream treated BALB/c mice (n = 5 per group). **(B)** Quantitative measurements of epidermal thickness are shown as means ± SEM. Student’s t-test ***p < 0.001. **(C)** Representative paraffin skin sections stained with anti-S100A8, anti-S100A9, IgG control and HE. One representative image for each condition is shown (original magnification 10 x, scale bar = 100 µm). **(D)** Serum concentrations of S100A8/S100A9 in mice was analyzed *via* ELISA after 7 days of IMQ treatment (▪) and controls (▼) (Student’s t-test **p < 0.01). **(E)** Differential expression pattern of S100A8, S100A9 and various cytokines on transcriptional level in IMQ (▪)- and placebo (▼)- treated skin sections. Transcripts were measured by quantitative real-time PCR and normalized to RPL housekeeping gene. Values are expressed as mean ± SEM (n ≥ 3 mice per group; Student’s t-test *p < 0.05).

To determine the level of S100A8 and S100A9 protein expression in epidermal keratinocytes during psoriasis-like inflammation, mice were topically treated with Aldara [imiquimod (IMQ)] for 7 days and the course of skin inflammation was monitored (**Figure 1A**). From day 3 onward, hallmarks of psoriasis-like dermatosis were visible. The back skin of mice started to show signs of erythema, scaling, and thickening, which continually increased in severity up to day 7 (**Figure 1A** IMQ). Mice treated with control cream did not show any signs of inflammation (**Figure 1A** placebo). HE staining of IMQ-treated back skin tissue sections showed significant thicker epidermis compared to sections obtained from placebo-treated mice including epidermal acanthosis and hyperproliferation of keratinocytes which is typical for psoriasis (**Figures 1B, C** and **Supplementary Figure 2**). Immunohistochemical staining revealed a strong increase of both proteins S100A8 and S100A9 in the skin after IMQ-treatment compared to control skin (**Figure 1C**; **Supplementary Figure 3C**). In addition to keratinocytes, leukocytes within the dermis showed a strong signal for S100A8 and S100A9 in skin sections of IMQ-treated mice, suggesting a strong infiltration of innate immune cells like neutrophils or inflammatory monocytes and CD4^+^CD17^+^ lymphocytes (**Supplementary Figures 3B, C**). Different inbred mice strains may show different inflammatory response patterns in the skin. We therefore compared BALB/c and C57BL/6 mice. However, there were no major differences between activity scores or cell composition between BALB/c and C57BL/6 mice (**Supplementary Figure 3**). We could also see a clear increase of S100A8/S100A9 complexes in serum of IMQ-treated mice detected by ELISA (**Figure 1D**). Quantification of messenger RNA (mRNA) expression of both proteins showed a parallel increase in back skin of IMQ-treated mice compared to controls (**Figure 1E**).

We next analyzed the mRNA expression patterns of certain inflammatory genes in the skin of mice. The transcriptional level of IL-17A was increased in parallel to the expression of S100A8 and S100A9 in the skin of IMQ-treated mice, but expression of IL-17F and IL-23 did not show any differences at mRNA level after 7 days compared to mice treated with control cream (**Figure 1E**). In addition, we did not see any difference in the expression patterns of cytokines of the IL-1-family like IL-1α, IL-1β, and IL-36, IL-6, TNFα between IMQ- and placebo-treated mice at mRNA level.

**Figure 2:**
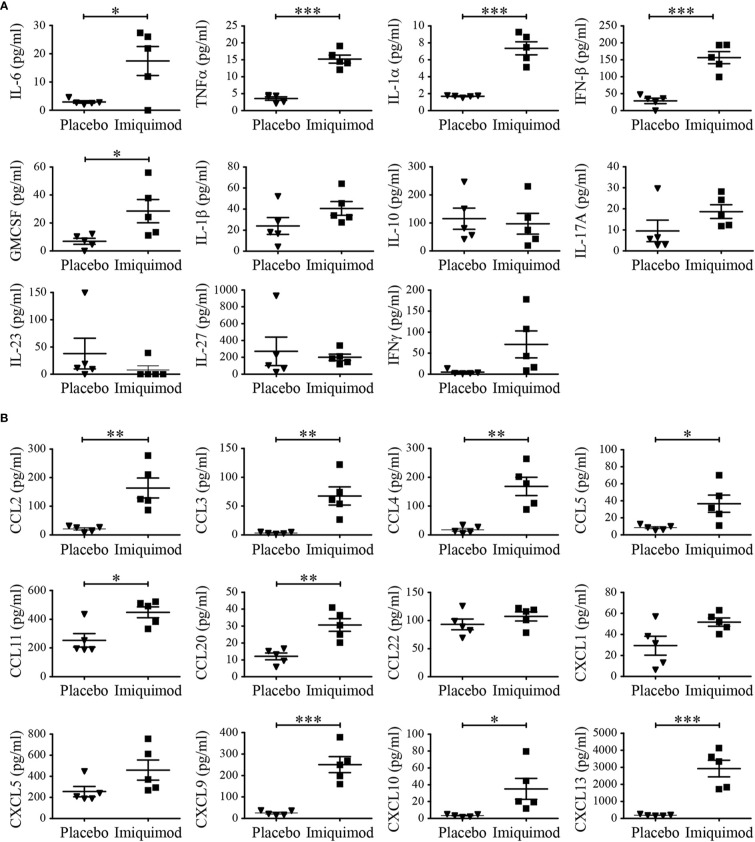
Imiquimod (IMQ) treatment induced inflammatory gene expression pattern. Naïve BALB/c mice were treated with IMQ or placebo cream for 7 consecutive days (n = 5 per group). **(A, B)** Cytokine/chemokines concentrations in serum of mice was analyzed at day 7 of IMQ (▪)- and placebo (▼)-treated mice with LEGENDplex multianalyte flow assay kit. In addition to individual data points, values are expressed as mean ± SEM. Student’s t-test *p < 0.05, **p < 0.01, ***p < 0.001.

In addition, serum of IMQ- and placebo-treated mice was analyzed by bead-based immunoassay for several cytokines/chemokines. Cytokines like IL-6, TNFα, IL-1α, IFN-β as well as GMCSF as well as pro-inflammatory chemokines as CCL2, CCL3, CCL4, CCL5, CCL11, CCL20, CXCL9, CXCL10 as well as CXCL13 could be detected in higher concentrations in serum of mice treated with IMQ compared to controls (**Figures 2A, B**). IL-17A showed the same trend but was not significant due to one outlier in the control group. IL-1β, IL-10, and IL-23 were not elevated in serum. CXCL22, CXCL1, and CXCL5 did not show any difference.

### Maturation of Keratinocytes Is Not Dependent on S100A8 and S100A9 Expression

**Figure 3:**
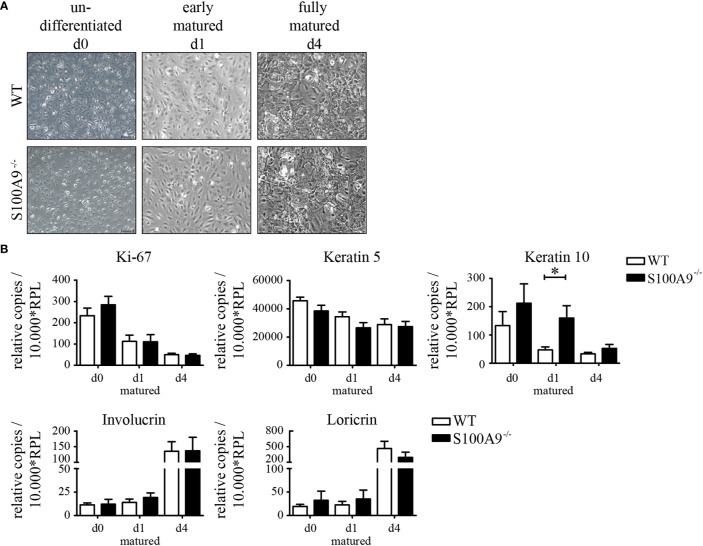
Morphology and differentiation of naïve primary keratinocytes of wildtype and S100A9^−/−^ mice. Primary epidermal keratinocytes were isolated from naïve wildtype C57BL/6 (WT)- and S100A9^−/−^ mice. **(A)** Representative phase contrast images of primary keratinocytes from WT- and S100A9^−/−^ mice at indicated stages of maturation (original magnification 10 x, scale bar = 100 µm). **(B)** Quantitative real-time PCR analysis of proliferation and maturation markers in keratinocytes from WT (white column)- and S100A9^−/−^ (black column) mice at indicated stages of maturation. Columns represent means ± SEM of at least eight independent experiments. Values are normalized to RPL housekeeping gene. Time course and cell culture conditions of primary keratinocytes are illustrated in **Supplementary Figure 1**. Student’s t-test *p < 0.05.

Because we could see a substantial increase of S100A8/S100A9 (**Figure 1**) in keratinocytes during inflammation we next looked for effects of S100A8/S100A9 expression in primary keratinocytes. We first compared proliferation and maturation of naïve keratinocytes from healthy WT- and S100A9^−/−^ C57BL/6 mice in cell culture. Both primary keratinocyte cell types showed the same morphology under basal conditions as well as similar morphological changes during early (d1 of maturation) and final maturation (d4 of maturation; **Figure 3A**). Undifferentiated cells (d0) are flat, smooth, and connected in a rather loose structure. After induction of maturation cells changed their morphology within hours (data not shown) and presented a more compact and cobblestone-like structure. The contacts between cells became denser and more solid (**Figure 3A**, d1). When keratinocytes were fully matured (d4 of maturation), they stratified and formed a cornified cell envelope, which is typical for differentiated keratinocytes of the stratum corneum [**Figure 3A**, fully matured (d4)].

It is known that patients with psoriasis show a hyperproliferation of keratinocytes and parakeratosis due to alterations of epidermal differentiation. To investigate a functional role of S100A8 and S100A9 during proliferation and maturation we investigated the expression levels of genes associated with proliferation and maturation in both WT- and S100A9^−/−^ keratinocytes. Analyzing expression of Ki-67 and keratin 5 as marker genes for proliferation we could not see a difference between keratinocytes isolated from WT or S100A9^−/−^ mice (**Figure 3B**). Only down-regulation of keratin 10 expression during maturation was delayed in S100A9^−/−^ keratinocytes on day 1 compared to WT cells but there were no differences at day 0 and day 4. Also genes typical for the granular layer of the skin like involucrin and loricrin showed no differences during maturation of WT or S100A9^−/−^ keratinocytes. S100A8/S100A9 does not appear to affect the maturation of keratinocytes in monolayer culture significantly.

**Figure 4:**
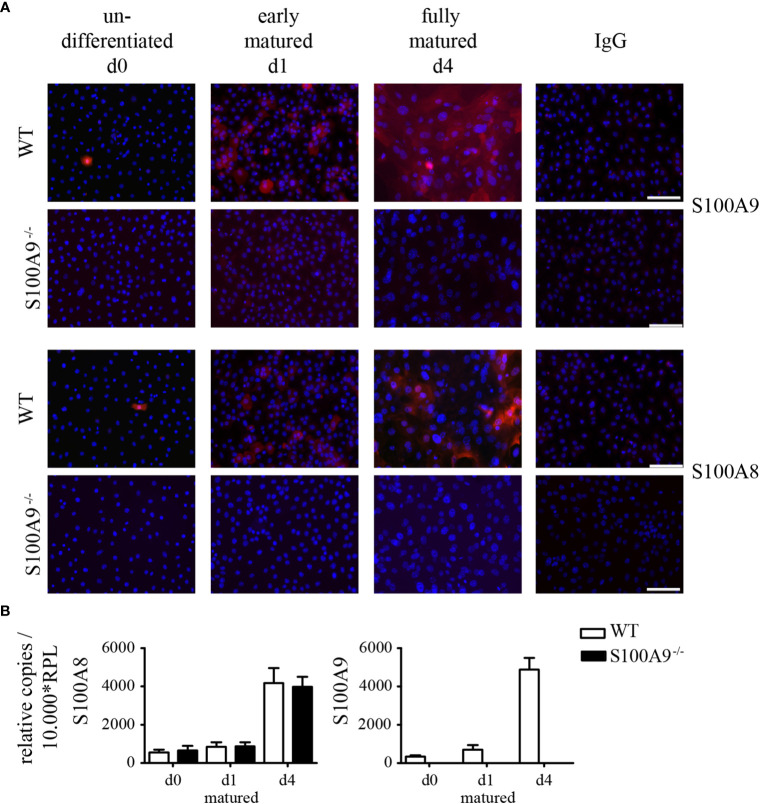
Expression of S100A8 and S100A9 in naïve primary keratinocytes of wildtype and S100A9^−/−^ mice. Primary keratinocytes were isolated from wildtype C57BL/6 (WT)- and S100A9^−/−^ mice and matured for indicated time points *ex vivo*. **(A)** Representative immunofluorescence images of cell nuclei (DAPI, blue), S100A8, S100A9, or IgG control (Alexa Fluor 546, red) in primary WT- and S100A9^−/−^ keratinocytes at indicated stages of maturation (original magnification 20 x, scale bar = 100 µm). **(B)** Transcriptional expression patterns of S100A8 and S100A9 were determined by quantitative real-time PCR analysis in primary WT (white column)- and S100A9^−/−^ (black column) keratinocytes at indicated stages of maturation. Columns represent the means ± SEM of at least eight independent experiments. Values are normalized to RPL housekeeping gene. Student’s t-test showed no significances.

We next investigated the expression of S100A8 and S100A9 proteins in cultured primary keratinocytes by immunofluorescence microscopy. In undifferentiated (d0) as well as early matured (d1) WT keratinocytes we could detect individual single positive cells expressing S100A8 and S100A9 at protein level (**Figure 4A**). Fully matured WT keratinocytes (d4), however, showed a strong signal for S100A8 and S100A9. Expression of both S100A8 and S100A9 proteins in these WT keratinocytes increased during maturation. As expected, keratinocytes obtained from S100A9^−/−^ mice did not show any immune signal for S100A9 protein (**Figure 4A**) or mRNA (**Figure 4B**). Knock-out of the S100A9 gene in mice lead also to a loss of S100A8 at protein level in myeloid cells (21). Accordingly, we could also not detect any S100A8 on protein level in S100A9^−/−^ keratinocytes although S100A8 mRNA expression during maturation was comparable to wildtype controls (**Figure 4B**). S100A8 and S100A9 form hetero-complexes. The depletion of S100A9 results in an instability of the S100A8 molecule, as well leading to protein degradation despite normal RNA values (6, 8, 30).

### S100A8 and S100A9 Expression Does Not Alter the Inflammatory Response Pattern of Keratinocytes

**Figure 5:**
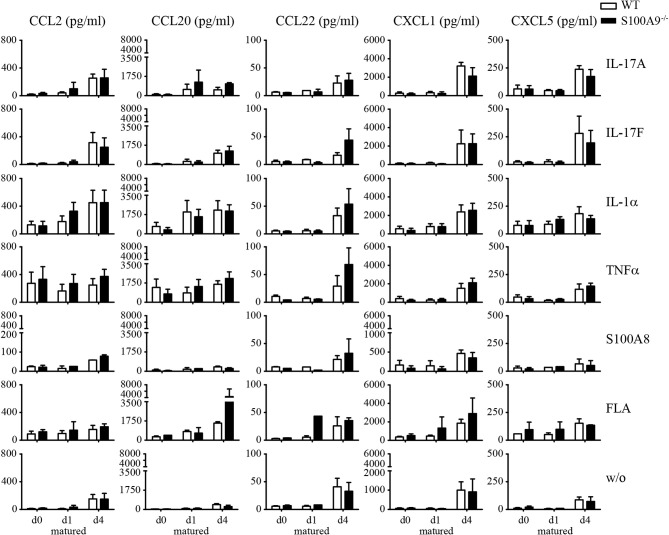
Inflammatory response patterns of primary keratinocytes of wildtype and S100A9^−/−^ mice. Primary keratinocytes were isolated from naïve wildtype C57BL/6 (WT, white column)- and S100A9^−/−^ (black column) mice, matured for indicated time points and stimulated for 24 h with indicated reagents [IL-17A, IL-17F, IL-1α, TNFα, S100A8, flagellin (FLA)]. Supernatants were collected for chemokine evaluation *via* flow cytometry associated bead-based immunoassay. Columns represent the means ± SEM of at least two independent experiments. Student’s t-test showed no significances between wildtype and S100A9^−/−^ mice.

To address the question if expression of S100A8 and S100A9 alters the inflammatory response pattern we compared keratinocytes isolated from either WT or S100A9^−/−^ mice. Cells were cultured in keratinocyte growth medium until they reached a confluency of approximately 70–80%. Subsequently keratinocytes were stimulated with inflammatory mediators at d0, d1, and d4 of maturation, e.g., under basal conditions or after induction of maturation with growth medium containing CaCl_2_. We chose stimuli known to be relevant in the pathogenesis of psoriasis like IL-17A, IL-17F, TNFα, IL-1α, flagellin as well as murine S100A8 as endogenous Toll-like receptor (TLR)-trigger. Naïve keratinocytes released only CCL2, CCL20, CCL22, CXCL1, CXCL5, and IL-1α after 24 h of stimulation (**Figure 5** and **Supplementary Figure 4**). Our stimuli induced a release of these cytokines or chemokines which was stronger in fully matured (d4) compared to early matured keratinocytes (d1). No remarkable difference in the inflammatory response pattern was detectable between keratinocytes isolated from WT and S100A9^−/−^ mice.

### S100A8 and S100A9 Expression Is Induced During Inflammatory Activation of Keratinocytes

**Figure 6:**
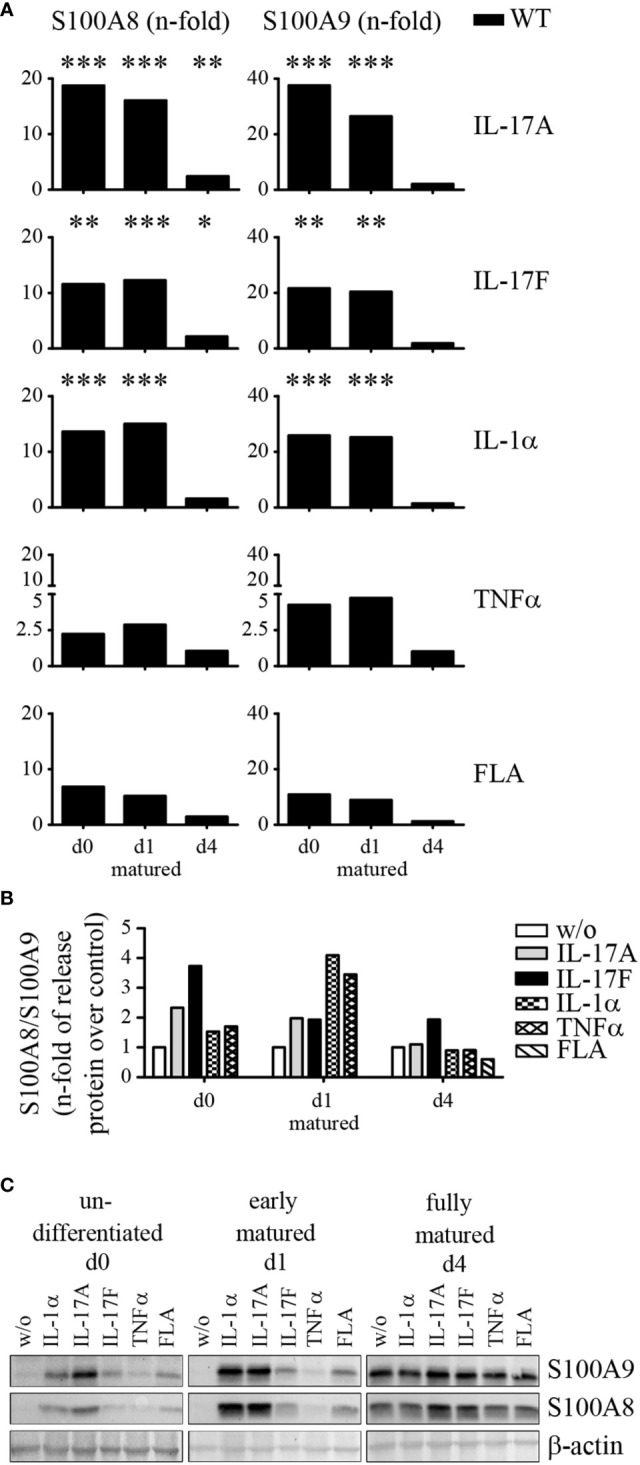
Stimulation of S100A8 and S100A9 protein expression in naïve primary keratinocytes. Primary keratinocytes were isolated from wildtype C57BL/6 mice, matured, and stimulated for 24 h *ex vivo*. **(A)** Messenger RNA (mRNA) expression of S100A8 and S100A9 were analyzed by quantitative real-time PCR. Columns represent means ± SEM of at least five independent experiments. Values were standardized to RPL housekeeping gene and represent the n-fold to their respective unstimulated WT keratinocytes at each maturation time point (w/o = 1). Asterisks indicate significant differences to unstimulated controls based on the original expression data (ANOVA *p < 0.05; **p < 0.01; ***p < 0.001). **(B)** Release of S100A8/S100A9 into culture supernatants by WT keratinocytes during maturation was analyzed after incubation with different stimuli *via* ELISA. Columns represent the means ± SEM of at least three independent experiments. Values represent the n-fold to unstimulated WT keratinocytes (w/o = 1). Student’s t-test revealed no significant differences. **(C)** Expression levels of S100A8 and S100A9 in primary WT keratinocytes were determined by Western blotting. β-actin served as loading control. One representative experiment out of two independent experiments is shown.

To investigate whether IL-17A, IL-17F, IL-1α, TNFα, or flagellin alter the expression of S100A8 and S100A9 primary murine keratinocytes were isolated, cultured, and matured as described above (**Supplementary Figure 1**). None of the used stimuli showed any influence on the proliferation of keratinocytes (data not shown). Quantitative real time PCR was performed to quantify mRNA expression of S100A8 and S100A9. In non-differentiated (d0) and early matured keratinocytes (d1) stimulation by IL-17A induced a strong up-regulation of S100A8 and S100A9 mRNA expression of up to 20 to 40 fold compared to control cells (**Figure 6A** and **Supplementary Figure 5**). In fully matured keratinocytes (d4) fold-changes of mRNA induction were much lower but protein levels were still increased, also in the absence of a stimulus (**Figure 6C**). Cells stimulated with IL-17F, IL-1α, and flagellin show a similar response in non-differentiated (d0) and early matured keratinocytes (d1) but only induced a 10 to 20 fold up-regulation of mRNA expression for S100A8 and S100A9, respectively. Stimulation with TNFα enhanced mRNA expression of S100A8 and S100A9 2 to 5 times in un-differentiated and early matured keratinocytes (d1). In addition, we could also see a clear release of S100A8/S100A9 complexes in supernatants of stimulated naïve keratinocytes *in vitro* compared to control cells (**Figure 6B**). Especially, IL-17A and IL-17F triggered naïve, undifferentiated (d0) keratinocytes to release S100A8/S100A9. Furthermore, IL-1α and TNFα stimulation led to a 3 to 4 fold up-regulation of S100A8/S100A9 in early matured keratinocytes (d1) compared to control cells. Fully matured keratinocytes (d4) exhibited only a 2 fold increase of S100A8/A9 protein release after IL-17F stimulation.

For analysis of protein expression, primary naïve keratinocytes were isolated, cultured, matured, and stimulated with IL-17A, IL-17F, IL-1α, TNFα, and flagellin for 24 h as described above. Cell lysates were analyzed for S100A8 and S100A9 proteins by western blot analysis using specific antibodies. Without any stimuli keratinocytes do not show a strong signal for S100A8 and S100A9 under basal conditions or during early maturation (d1) (**Figure 6C**). In contrast, fully matured keratinocytes (d4) showed already a strong protein signal for both S100A8 and S100A9 in control cells as already shown by immunofluorescence microscopy above. In undifferentiated keratinocytes stimulation with IL-17A and IL-1α induced the strongest up-regulation of S100A8 and S100A9 protein expression. IL-17F and flagellin also induced S100A8 and S100A9 protein expression albeit to a lesser extent. The lowest induction could be detected for TNFα. Early matured keratinocytes (d1) showed an almost identical response pattern to undifferentiated keratinocytes but on an overall higher basal level of S100A8 and S100A9 protein expression. Neither IL-17A, IL-17F, IL-1α, TNFα nor flagellin induced a significant up-regulation of the already high protein expression levels in fully matured keratinocytes (d4) compared to unstimulated controls.

### Human Gene Expression Analysis

**Figure 7:**
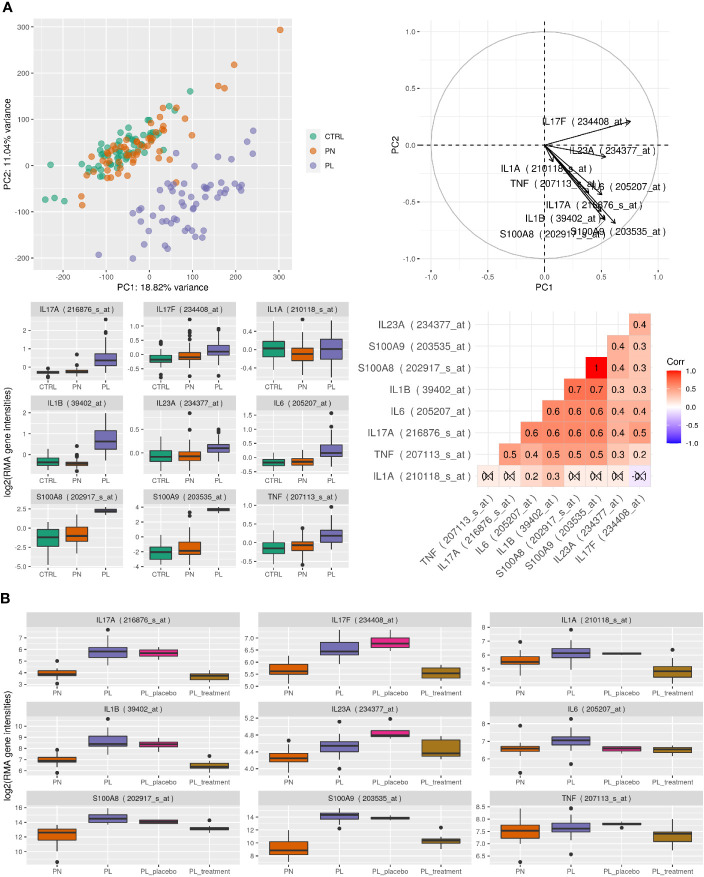
Human gene expression analysis. **(A)** Microarray expression data from psoriatic patients and normal controls. Top left: principle component analysis (PCA) of 180 samples with skin samples of healthy controls (CTRL, n = 64) and 58 paired samples of psoriasis patients (PN = uninvolved skin, PL = involved skin). Top right: contributions of selected gene probes toward the variance in first and second principle components from PCA. Bottom left: boxplot of selected gene probes in the separate groups. Bottom right: Spearman correlation coefficient of expression between selected gene probes, where correlations were crossed out if they were not found to be significant with p <0.05. Data taken from GEO accession ID GSE13355. **(B)** Microarray expression data from psoriatic patients during IL-17 directed treatment. The different groups represent 25 patients with lesional (PL) and non-lesional (PN) skin. Additionally, biopsies were taken of lesional skin after 43 days of placebo (PL_placebo) or 700 mg of brodalumab treatment (PL_treatment). Data were taken from GEO accession ID GSE53552.Boxplot of selected gene probes based on expression data run on Affymetrix HU133 Plus 2.0 microarrays.

We next analyzed published microarray expression data (GEO accession ID GSE13355) comparing paired samples of lesional and non-lesional skin from psoriatic patients and normal controls (**Figure 7A**). Performing principle component analysis (PCA) of samples from 64 controls and 58 paired samples of lesional and non-lesional skin from psoriasis patients showed a clear separation of lesional skin samples representing active inflammation. In addition, S100A8 and S100A9 were induced in lesional skin compared to non-lesional and control samples and differences of expression of S100A8 and S100A9 contributed toward the variance in first and second principle components from PCA comparable to genes known to be relevant in the pathogenesis of psoriasis like IL-17A, IL-23A, IL-1A, or TNF. S100A9 was the most significantly up-regulated gene (S100A8 number 12 of more than 500 significantly regulated genes) between controls or non-lesional and lesional psoriatic skin (p value for all comparisons after correction for multiple testing < 0.0001). Induction of S100A8 and S100A9 expression showed a significant correlation to the expression of IL-17A, IL-23A, IL-1B, or TNF (**Figure 7A**). We next analyzed the expression patterns of S100A8 and S100A9 in 25 paired samples of psoriasis treated with IL-17 directed therapy (700 mg of brodalumab) or placebo (GEO accession ID GSE53552). Expression of both S100-alarmins decreased after treatment comparable to IL-17A, IL-23A, and IL-1B (**Figure 7B**).

## Discussion

Throughout inflammatory dermatoses, especially in psoriasis, both S100A8 and S100A9 are highly up-regulated in skin lesions of patients (18). In addition, regulatory effects on cell dynamics and modulation of cytoskeletal proteins have been described for S100A8 and S100A9 in epithelial cells (31–33). However, none of these tasks has been directly addressed in primary keratinocytes. Comparing primary keratinocytes from mice lacking S100 expression with wildtype controls we found some unexpected results. In contrast to the widespread assumption our studies show that S100A8 and S100A9 do neither alter the differentiation status nor the inflammatory response pattern of keratinocytes *in vitro* but S100A8 and S100A9 represent rather additional inflammatory factors during IL-17-mediated dermal inflammation.

In line with previous reports, we found a high expression of both proteins S100A8 and S100A9 in a murine model of IMQ-induced psoriasis (16, 34) which confirms also data obtained from human psoriatic lesions showing a strong increase of S100A8 and S100A9 expression in keratinocytes (35). We found an abundant expression of both proteins in each epidermal layer and in infiltrated immune cells of the dermis as well as elevated S100-levels in serum of IMQ-treated mice compared to placebo-treated controls. Induction of S100 expression in the skin of IMQ-treated mice was associated with an increased transcriptional level of IL-17A and a number of inflammatory cyto- and chemokines. These cytokines, like TNFα, a cytokine critical for the initiation of psoriasis (36), and chemokines have functional roles in the inflammatory process of psoriasis. IL-6 is significantly up-regulated during psoriasis and it is known that keratinocytes as well as monocytes and neutrophils produce IL-6 during psoriatic inflammation (37). IL-6 is known to act synergistically with IL-1 and TNFα and shows the highest induction of gene expression in monocytes after S100-stimulation using transcriptomic analysis (9, 37, 38). Blocking TNFα is a highly effective therapy for psoriasis patients (39). The release of this protein by leukocytes or keratinocytes in turn activates an inflammatory cascade by stimulating cells like T_H_17 cells to produce psoriasis-promoting cytokines (40–43). In addition, IFN-β and GMCSF modulate leukocyte recruitment and cytokine production during IMQ-induced psoriasis (44, 45). CCL2 and CCL20 recruit dendritic cells, whereas CCL4 modulates chemotaxis of T_H_1 lymphocytes, dendritic cells as well as monocytes to the skin (46). CCL5 secreted by keratinocytes is involved in recruiting memory T cells and activated naive T cells and macrophages to psoriatic skin (47, 48). CXCL10 was detected in dermal infiltrates and keratinocytes isolated from active psoriatic plaques (49).

Serum concentrations of S100A8/S100A9 are a reliable biomarker for monitoring disease activity in psoriasis but also rheumatoid arthritis, psoriatic arthritis or infections underlining their clinical relevance (5, 15, 18, 50). Parallel with the expression of S100A8 and S100A9 IMQ-treated mice developed psoriasis features like erythema, scaling, thickening of skin, parakeratosis, acanthosis of the epidermis, signs of hyperproliferation of keratinocytes, and increased numbers of basal keratinocytes. These results indicate an association of S100 expression with altered keratinocyte proliferation and differentiation during psoriasis as suggested previously by different investigators (15, 51, 52). However, none of these published studies has functionally analyzed the consequences of S100A9 deletion in keratinocytes *in vitro*. In addition, one has to keep in mind that the model of IMQ-induced inflammation has also some caveats. For example, the mode of action of IMQ inducing skin inflammation is not fully understood and the inflammatory and histological changes do only represent some of the characteristics of human psoriasis (53).

The functional relevance of S100-alarmin expression and activity has been demonstrated in different models of psoriasis-like inflammation in mice *in vivo* by independent groups (6, 15). However, direct effects of S100A8 and S100A9 expression in keratinocytes have not been described so far. We therefore compared primary keratinocytes of wildtype and S100A9^−/−^ mice to detect relevant effects of these proteins in this cell type. S100A8^−/−^ are not available since S100A8^−/−^ mice are not viable and show an embryonic lethal phenotype. However, S100A9^−/−^ keratinocytes lack S100A8 at protein level as well since S100A8 is not stable in the absence of its binding partner. Neither undifferentiated nor matured naïve keratinocytes obtained from S100A9^−/−^ mice showed a different morphology compared to keratinocytes isolated from wildtype mice. Keratinocytes of both genotypes developed a compact and cobblestone-like structure, stratified, and formed a cornified cell envelope after fully maturation. Also analysis of gene expression of typical proliferation and maturation proteins like Ki-67, keratin 5, involucrin, and loricrin did not show any relevant differences between keratinocytes isolated from both mouse strains. Only down-regulation of keratin 10 showed some delay in the absence of S100A9 during early keratinocyte maturation. All other markers, proliferation, monolayer formation, and morphology showed no difference between wildtype and S100A9^−/−^ keratinocytes. In addition, S100A9^−/−^ mice showed no aberrant keratinocyte phenotype under steady state conditions *in vivo*. A compensatory role of S100A8 in S100A9^−/−^ keratinocytes can be excluded since S100A9^−/−^ cells lack expression of S100A8 at protein level as shown for leukocytes before (21, 30). Our results strongly indicate that S100A8 and S100A9 are no essential mediators of maturation or morphology in keratinocytes. However, one has to keep in mind, that we used a monolayer culture model *in vitro* which may differ from 3D culture conditions and therefore differences of wildtype and S100A9^−/−^ keratinocytes in the complex context *in vivo* cannot be finally excluded with our *in vitro* model.

Expression of S100A8 and S100A9 in keratinocytes is closely associated with markers of inflammation in murine and human psoriasis and keratinocytes are essential cellular mediators for the initiation and maintenance of this disease by secreting leukocyte-attracting chemokines like CXCL1, CCL20, and other inflammatory molecules like TNFα or IL-6 (1, 54). We therefore analyzed whether expression of S100A8 and S100A9 modulates the inflammatory response pattern of these keratinocytes. We compared primary keratinocytes obtained from WT or S100A9^−/−^ mice after stimulation with IL-17A, IL-17F, IL-1α, TNF, or flagellin. All of these inflammatory stimuli induced a significant inflammatory response most pronounced in terminally matured keratinocytes, but we found no difference between WT and S100A9^−/−^ keratinocytes. These results exclude a pivotal role of S100A8/S100A9 expression for the inflammatory response of keratinocytes.

However, looking on the effects of cytokines highly relevant in the pathogenesis of psoriasis, i.e., IL-17A, IL-17F, IL-1α, or TNF, on S100A8 or S100A9 expression in keratinocytes we found strong effects on mRNA induction for both genes most pronounced for IL-17A > IL-17F > IL-1α > TNF. Interestingly, in contrast to all other cytokines and chemokines investigated in our study induction of S100A8 and S100A9 expression was most impressive in early maturation stages of keratinocytes which points to the fact that expression of these alarmins reflects a specific inflammatory response pattern of keratinocytes. The biological relevance for inflammatory processes in the skin *in vivo*, however, is not finally proven by these results obtained *in vitro*. The general relevance of S100A8/S100A9 expression during psoriasis is confirmed by the fact that inflammation in psoriasis-like skin disease in mice is strongly attenuated after deletion of the S100A9 gene in mice (15). Transcriptome analysis comparing gene expression in control samples, lesional and non-lesional skin of psoriasis patients and a clear down-regulation of S100A8 and S100A9 expression during response to IL-17 directed therapy also points to a relevant role of pro-inflammatory S100-alarmins in the course of psoriasis.

S100A8 and S100A9 have manifold inflammatory activities, which may amplify disease activity in psoriasis. Dimers of S100A8/S100A9 bind to TLR4 and induce an inflammatory response in phagocytes *via* transcriptional activation of NF-κB (6, 9). In addition, expression of S100A8/S100A9 links innate immunity and auto-immune responses. In a murine dermatitis model S100A8/S100A9 expression drives development of auto-reactive CD8^+^ T cells mediated by increased expression of IL-17 (13). Interestingly, TNF, IL-17, IL-1, and S100A8/S100A9 have been described to form a positive feedback network driving disease activity in murine models of auto-immune arthritis or systemic lupus erythematosus (13, 55). The question whether keratinocytes are the main source of pro-inflammatory S100-alarmins in the course of psoriasis cannot be answered by published data or our present study but has to be addressed in mouse strains with tissue specific deletions of these proteins. Beside keratinocytes, monocytes and neutrophils are potential cellular sources for release of significant amounts of S100-alarmins at the site of cutaneous inflammation like psoriasis (4, 5, 7).

Taken together our data indicates that the expression of S100A8 and S100A9 does not primarily influence differentiation or inflammatory activation of keratinocytes but represents a pro-inflammatory factor released by these cells in the context of the dermal innate immune response. High abundance of these S100-proteins during psoriasis can be explained especially by the effects of IL-17A, IL-17F, TNF, and IL-1α on S100A8/S100A9 expression, which are all central players in the pathogenesis of psoriasis. We have recently demonstrated that the inflammatory activity of S100A8/S100A9 is restricted to local sites of inflammation due to an auto-inhibitory mechanism. Calcium induced S100-tetramer formation blocks specific peptide sequences which otherwise activate TLR4 (6). Blocking release or inflammatory effects of S100A8/S100A9 may be a promising strategy for future therapeutic approaches to locally treat psoriatic patients.

## Data Availability Statement

The original contributions presented in the study are included in the article/**Supplementary Material**. Further inquiries can be directed to the corresponding author.

## Ethics Statement

The animal study was reviewed and approved by Landesamt für Umwelt, Natur, und Verbrauchershutz NRW.

## Author Contributions

CC, SZ, KL, and JR conceived and designed the experiments. CC, SZ, KL, and JH performed the experiments. CC, SZ, LM, JH, TV, and JR analyzed the data. CC, SZ, and JR wrote the manuscript. All authors contributed to the article and approved the submitted version.

## Funding

This study was funded by grants of the Interdisciplinary Center of Clinical Research at the University of Muenster (Ro2/023/19), the German Research Foundation CRC 1009 B07, B08, B09 and Z2, and RO 1190/14-1 to JR, TV, and KL.

## Conflict of Interest

The authors declare that the research was conducted in the absence of any commercial or financial relationships that could be construed as a potential conflict of interest.

## Supplementary Material

Supplementary Figure 1 Schematic diagram of the differentiation and stimulation steps of primary keratinocytes *in vitro*. Primary naïve keratinocytes were isolated from mice tails and cultured on coated culture plates in normal growth medium (Keratinocyte Growth Medium 2 with supplement mix; KGM) with 0.06 mM CaCl_2_ to a confluence of 70% to 80%. Afterward, the medium was changed to 1 mM CaCl_2_ to initiate the maturation of keratinocytes. Depending on the maturation status, keratinocytes were stimulated for 24 h in cell culture medium (Keratinocyte Growth Medium 2 w/o supplement mix (KBM) with 1 mM CaCl_2_) with flagellin from S. typhimurium (100 ng/ml), rm IL-1α, rm IL-17A, rm IL-17F and rm TNFα (all 100 ng/ml) and S100A8 (5 µg/ml). d0 = undifferentiated: KBM media with 0.06 mM CaCl_2_ after 24 h stimulation; d1 = early matured: KBM media with 1 mM CaCl_2_ after 24 h stimulation; d4 = fully matured: KGM media with 1 mM CaCl_2_ for 72 h and afterwards KBM media with 1 mM CaCl_2_ for 24 h stimulation.

Supplementary Figure 2 Measurement of epidermal thickness in IMQ-treated mice. Representative image illustrating the measurement of epidermal thickness (white bars, original magnification 10 x, scale bar = 100 µm)

Supplementary Figure 3 Comparison of IMQ treatment in C57BL/6 and BALB/c mice. Naïve C57BL/6- or BALB/c mice were treated with IMQ or placebo cream for 7 consecutive days. (A) The cumulative PASI score (erythema + scaling + thickness) of the skin lesion in IMQ-induced psoriasis-like mice on day 7 are shown. Data are presented as the mean ± SEM (n = 5 per group). Students`s t-test did not reveal significant differences. (B) Quantification of the cellular composition in placebo or IMQ-treated skin sections is shown. Data are presented as the mean ± SEM (n = 5 per group). Students`s t-test did not reveal significant differences between mouse strains. Area for cell counting = 100 µm x 100 µm. (C) Representative immunofluorescence images of S100A9, S100A8, Gr1, MPO and cytokeratin staining of placebo or IMQ-induced psoriatic skin sections of C57BL/6 or BALB/c mice on day 7 (original magnification: 200 x; Gr1/MPO: 100x; scale bar = 50 µm). Alexa Fluor 488: green, Alexa Fluor 549: red, cell nuclei DAPI: blue. One representative of at least 2 separate experiments is shown.

Supplementary Figure 4 Cytokine associated inflammatory response of primary wildtype and S100A9^−/−^ keratinocytes. Primary keratinocytes were isolated from naïve wildtype C57BL/6 (WT, white column)- and S100A9^−/−^ (black column) mice, matured for indicating time points and stimulated for 24 h with indicated reagents (IL-17A, IL-17F, IL-1α, TNFα, S100A8, flagellin (FLA)). Supernatants were collected for IL-1α evaluation *via* flow cytometry associated bead-based immunoassay. Columns represent the means ± SEM of at least 2 independent experiments. Students`s t-test did not reveal significant differences.

Supplementary Figure 5 Induction of S100A8 and S100A9 protein expression in naïve primary BALB/c keratinocytes. Primary epidermal keratinocytes were isolated from wildtype BALB/c mice, matured and stimulated for 24 h *ex vivo*. mRNA expression of S100A8 and S100A9 were analyzed by quantitative real-time PCR. Columns represent means of two independent experiments. Values were standardized to RPL housekeeping gene and represent the n-fold to their respective unstimulated WT keratinocytes at d0 and d1 of maturation (w/o = 1).

Supplementary Table 1 Primer pairs used for Real Time-qPCR analysis.
