# Supplementary figures and images for: Interleukin 17 Promotes Expression of Alarmins S100A8 and S100A9 During the Inflammatory Response of Keratinocytes
Source: Front Immunol. 2021 Feb 12;11:599947. doi: 10.3389/fimmu.2020.599947 (PMC7906991; doi:10.3389/fimmu.2020.599947)

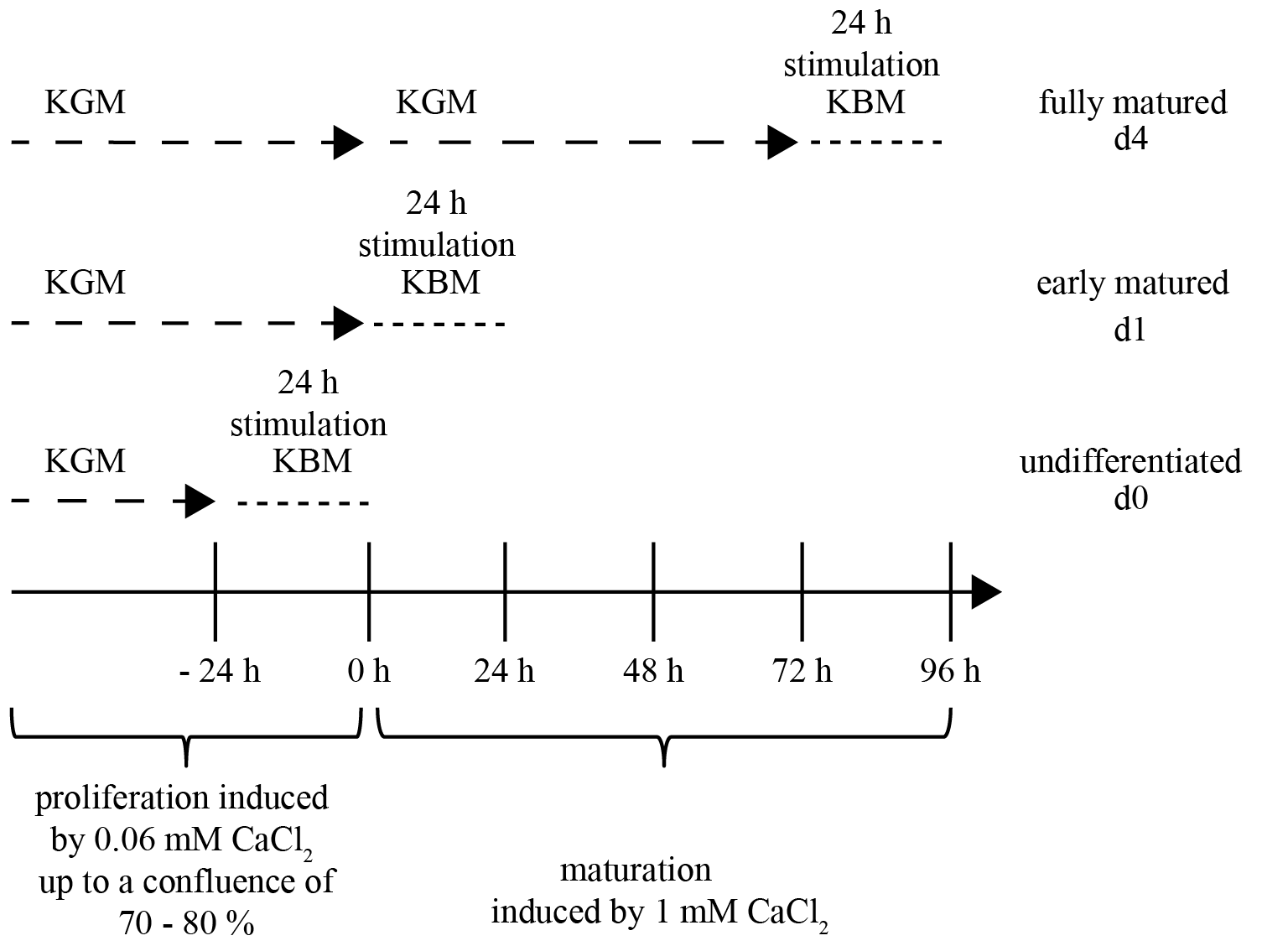

Supplement: Supplementary Figure 1 — Schematic diagram of the differentiation and stimulation steps of primary keratinocytes in vitro. Primary naïve keratinocytes were isolated from mice tails and cultured on coated culture plates in normal growth medium (Keratinocyte Growth Medium 2 with supplement mix; KGM) with 0.06 mM CaCl2 to a confluence of 70% to 80%. Afterward, the medium was changed to 1 mM CaCl2 to initiate the maturation of keratinocytes. Depending on the maturation status, keratinocytes were stimulated for 24 h in cell culture medium (Keratinocyte Growth Medium 2 w/o supplement mix (KBM) with 1 mM CaCl2) with flagellin from S. typhimurium (100 ng/ml), rm IL-1α, rm IL-17A, rm IL-17F and rm TNFα (all 100 ng/ml) and S100A8 (5 µg/ml). d0 = undifferentiated: KBM media with 0.06 mM CaCl2 after 24 h stimulation; d1 = early matured: KBM media with 1 mM CaCl2 after 24 h stimulation; d4 = fully matured: KGM media with 1 mM CaCl2 for 72 h and afterwards KBM media with 1 mM CaCl2 for 24 h stimulation. [file Image_1.tif]

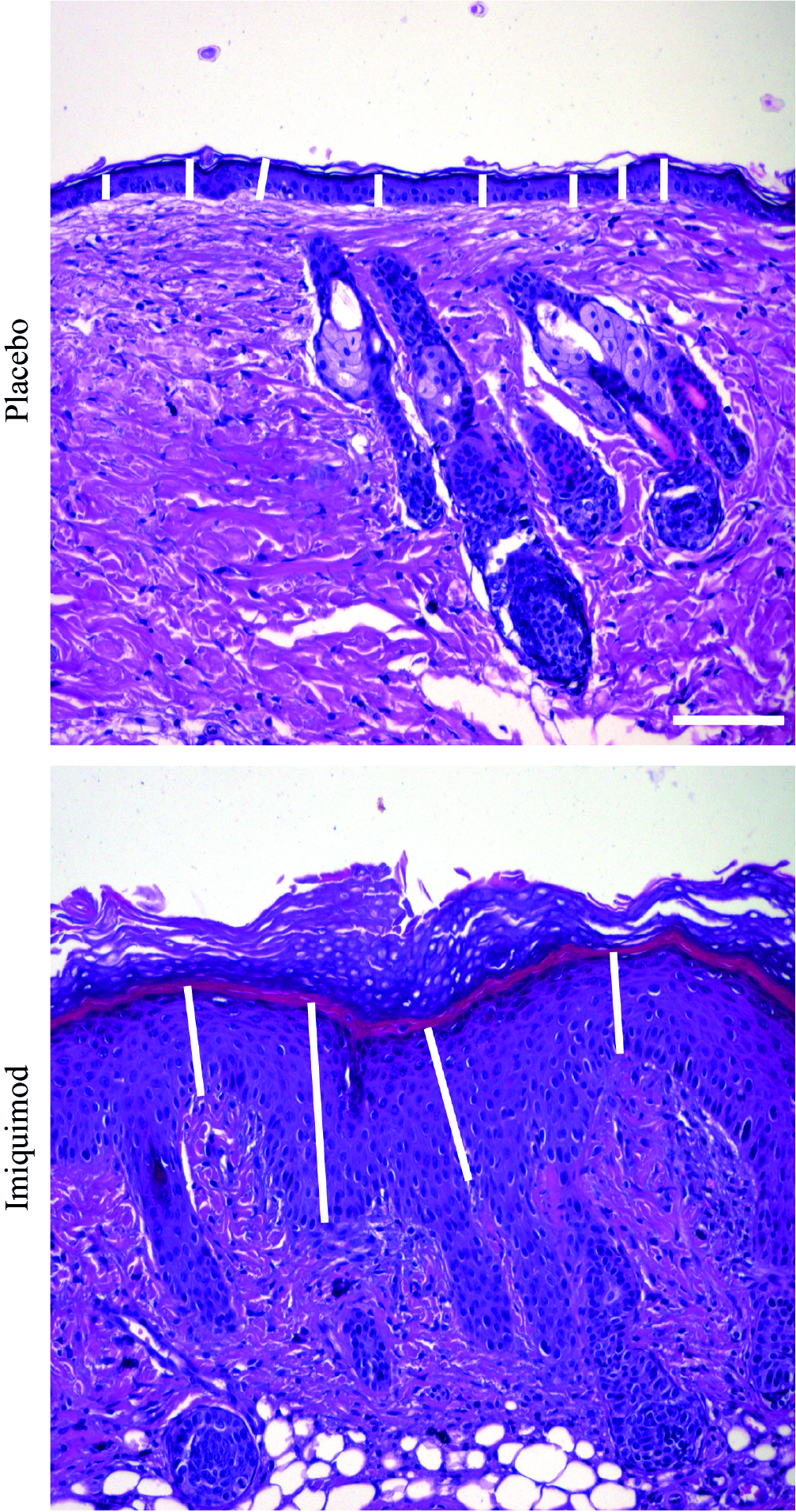

Supplement: Supplementary Figure 2 — Measurement of epidermal thickness in IMQ-treated mice. Representative image illustrating the measurement of epidermal thickness (white bars, original magnification 10 x, scale bar = 100 µm) [file Image_2.tif]

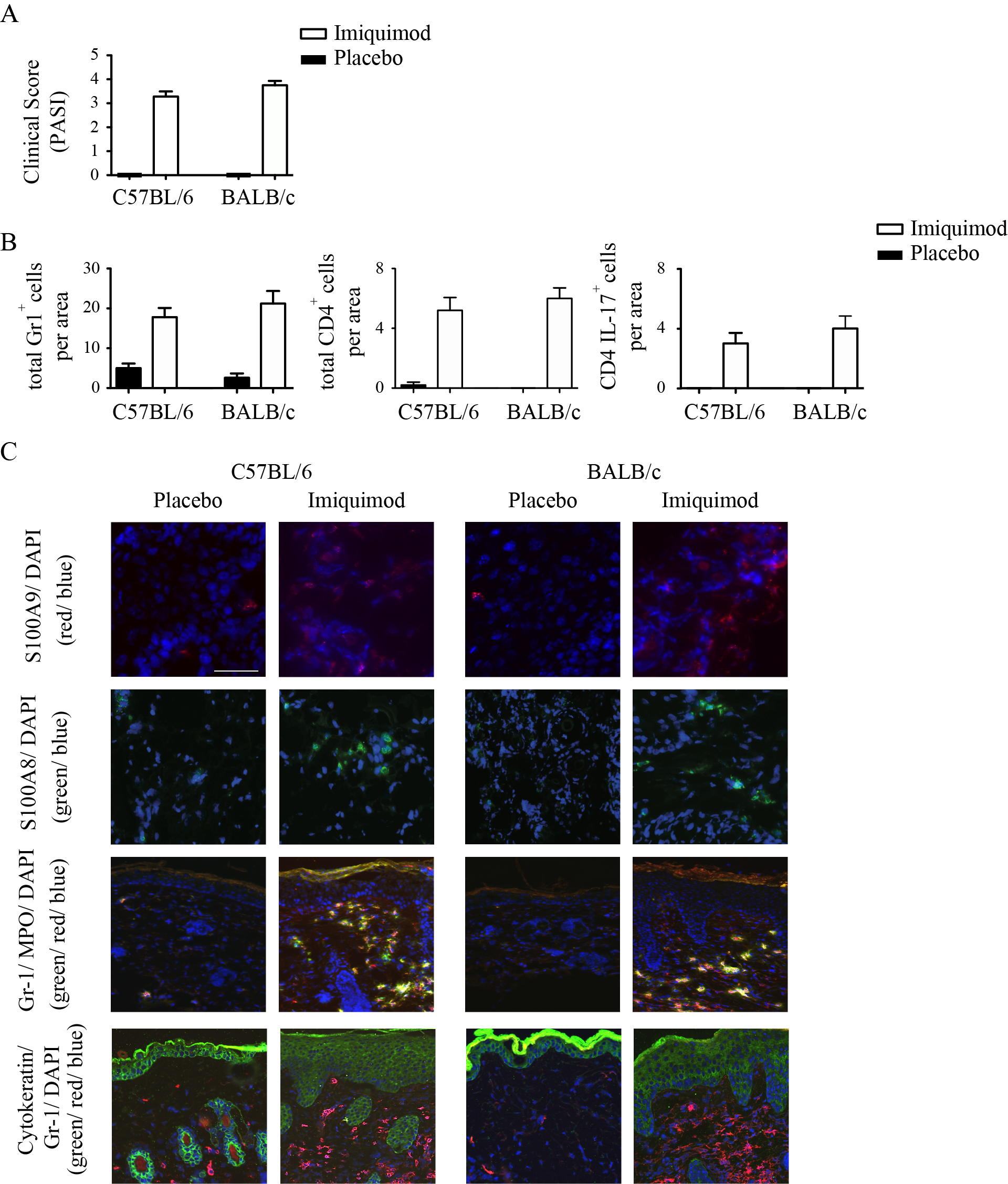

Supplement: Supplementary Figure 3 — Comparison of IMQ treatment in C57BL/6 and BALB/c mice. Naïve C57BL/6- or BALB/c mice were treated with IMQ or placebo cream for 7 consecutive days. (A) The cumulative PASI score (erythema + scaling + thickness) of the skin lesion in IMQ-induced psoriasis-like mice on day 7 are shown. Data are presented as the mean ± SEM (n = 5 per group). Students`s t-test did not reveal significant differences. (B) Quantification of the cellular composition in placebo or IMQ-treated skin sections is shown. Data are presented as the mean ± SEM (n = 5 per group). Students`s t-test did not reveal significant differences between mouse strains. Area for cell counting = 100 µm x 100 µm. (C) Representative immunofluorescence images of S100A9, S100A8, Gr1, MPO and cytokeratin staining of placebo or IMQ-induced psoriatic skin sections of C57BL/6 or BALB/c mice on day 7 (original magnification: 200 x; Gr1/MPO: 100x; scale bar = 50 µm). Alexa Fluor 488: green, Alexa Fluor 549: red, cell nuclei DAPI: blue. One representative of at least 2 separate experiments is shown. [file Image_3.tif]

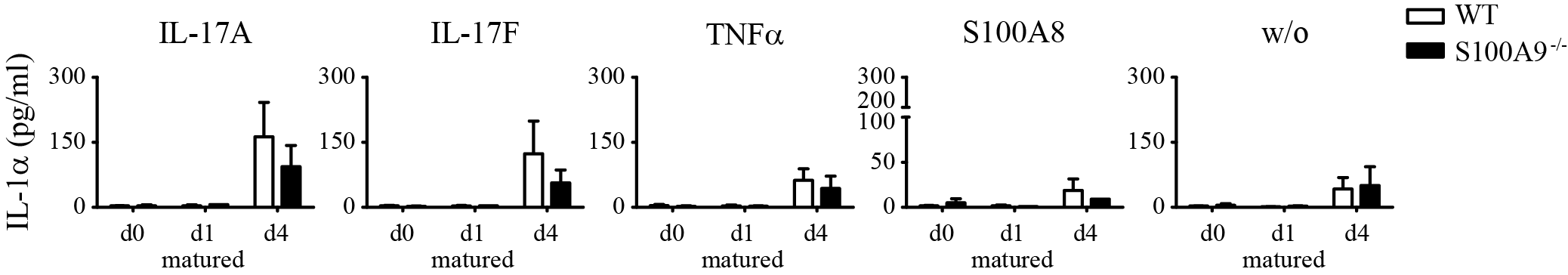

Supplement: Supplementary Figure 4 — Cytokine associated inflammatory response of primary wildtype and S100A9−/− keratinocytes. Primary keratinocytes were isolated from naïve wildtype C57BL/6 (WT, white column)- and S100A9−/− (black column) mice, matured for indicating time points and stimulated for 24 h with indicated reagents (IL-17A, IL-17F, IL-1α, TNFα, S100A8, flagellin (FLA)). Supernatants were collected for IL-1α evaluation via flow cytometry associated bead-based immunoassay. Columns represent the means ± SEM of at least 2 independent experiments. Students`s t-test did not reveal significant differences. [file Image_4.tif]

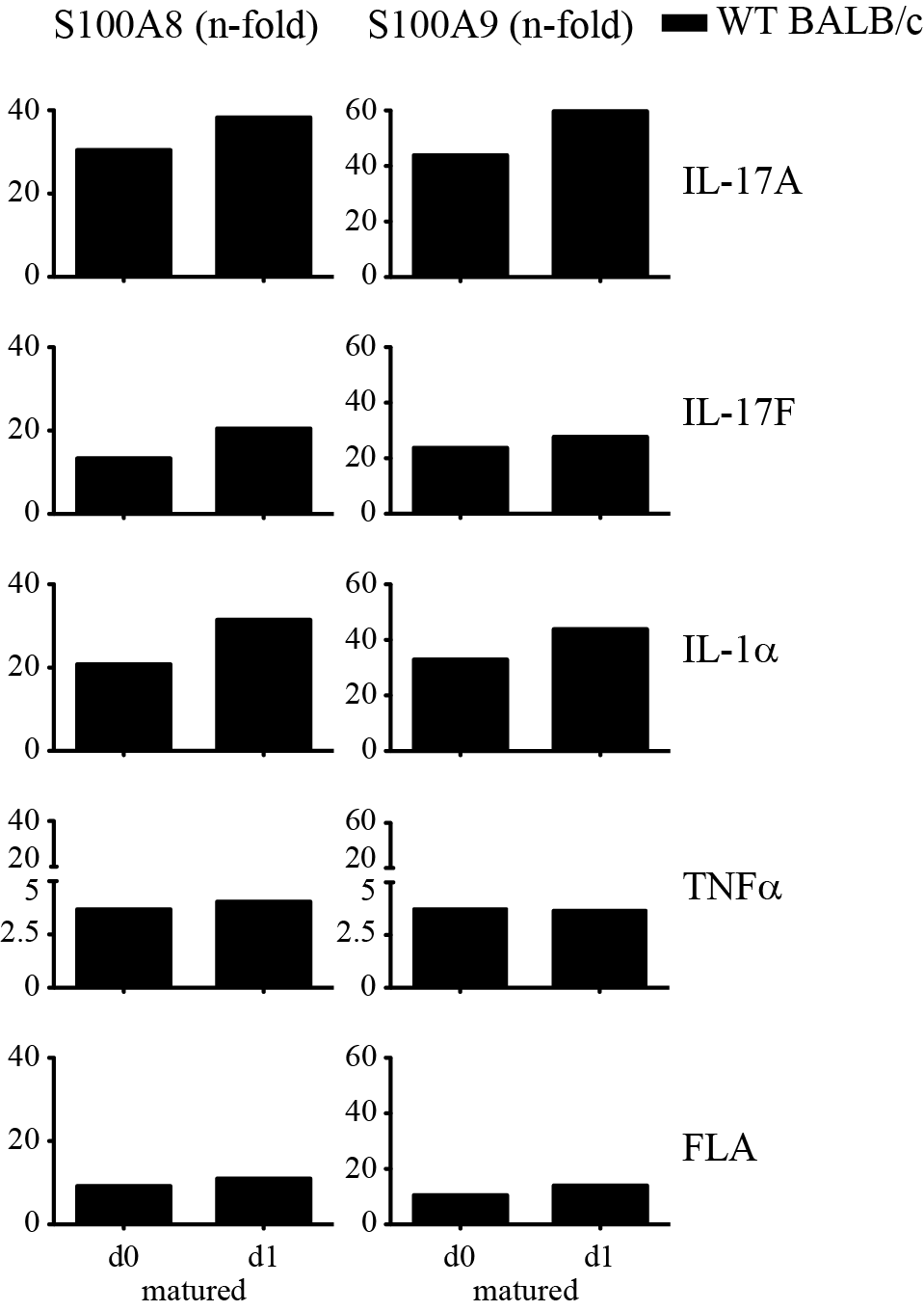

Supplement: Supplementary Figure 5 — Induction of S100A8 and S100A9 protein expression in naïve primary BALB/c keratinocytes. Primary epidermal keratinocytes were isolated from wildtype BALB/c mice, matured and stimulated for 24 h ex vivo. mRNA expression of S100A8 and S100A9 were analyzed by quantitative real-time PCR. Columns represent means of two independent experiments. Values were standardized to RPL housekeeping gene and represent the n-fold to their respective unstimulated WT keratinocytes at d0 and d1 of maturation (w/o = 1). [file Image_5.tif]
