# Supplementary material for: Interleukin 17 Promotes Expression of Alarmins S100A8 and S100A9 During the Inflammatory Response of Keratinocytes
Source: Front Immunol. 2021 Feb 12;11:599947. doi: 10.3389/fimmu.2020.599947 (PMC7906991; doi:10.3389/fimmu.2020.599947)
Supplement: Supplementary Table 1 — Primer pairs used for Real Time-qPCR analysis. [file Table_1.docx]

Table 1: Primer pairs used for Real Time-qPCR analysis

|  | Primer pair – murine - 5`- 3` |
| --- | --- |
| RPL | 5`-TGG TCC CTG CTG CTC TCA AG-3` 5`-GGC CTT TTC CTT CCG TTT CTC-3` |
| S100A8 | 5`-ATC ACC ATG CCC TCT ACA AGA ATG-3` 5`-GTC CAA TTC TCT GAA CAA GTT TTC G-3` |
| S100A9 | 5`-CTC TAG GAA GGA AGG ACA CC-3` 5´-GCC ATC AGC ATC ATA CAC TC-3` |
| IL-6 | 5`-TGA GAT CTA CTC GGC AAA CCT AGT G-3` 5`-CTT CGT AGA GAA CAA CAT AAG TCA GAT ACC-3` |
| TNF-alpha | 5`-AGA AAC ACA AGA TGC TGG GAC AGT-3` 5`-CCT TTGCAG AAC TCA GGAATG G-3` |
| IL-36alpha | 5`-TAC ATG GGA GTG CAA AGG CC-3` 5`-AGT GGG CAG CTC CCT TTA GAG C-3` |
| IL-17A | 5`-CAG AAG GCC CTC AGA CTA CC-3` 5`-TCT CGA CCC TGA AAG TGA AG-3` |
| IL-17F | 5`-CTC TGT GTG AAG GCC GAT CTC-3` 5`-TGC CAT GCA CAC CTT ACT GAG-3` |
| IL-1alpha | 5`-TGT TGC AGG TCA TTT AAC CAA GTG-3` 5-CAC CTT ACA CCT ACC AGA GTG ATT TG-3` |
| IL-1beta | 5`-TGT CTT GGC CGA GGA CTA AGG-3` 5`-TGG GCT GGA CTG TTT CTA ATG C-3` |
| IL-23 | 5`-ATC CAG TGT GAA GAT GGT TGT GA-3` 5`-CTG GAG GAG TTG GCT GAG TC-3` |
| IFN-gamma | 5`-TGC TGA TGG GAG GAG ATG TCT AC-3` 5`-TTT CTT TCA GGG ACA GCC TGT TAC-3` |
| Keratin 5 | 5`-CAG GCC ACG TTT CCT TTT TCT-3` 5`-TGC TGA CGT GCA ACA CAA ATG-3` |
| Keratin 10 | 5`-ATT ATT GAG GAG GTG ACA CCT GAG G-3` 5`-CCCGTA TGA AGA GAC TCT TCT ATG CA-3` |
| Ki-67 | 5`-GAC AGC TTC CAA AGC TCA CC-3` 5`-TGT GTC CTT AGC TGC CTC CT-3` |
| Loricrin | 5`-GTT GCA ACG GAG ACA ACA GAG C-3` 5`-CAG GAT ACA CCT TGA GCG ACT CA-3` |
| Involucrin | 5`-TCA GAC CAG CCA CTG GAT CAA-3` 5`- GTG TCC GGT TCT CCA ATT CGT-3´ |
